# Tumor necrosis factor reduces *Plasmodium falciparum* growth and activates calcium signaling in human malaria parasites

**DOI:** 10.1016/j.bbagen.2016.04.003

**Published:** 2016-07

**Authors:** Laura N. Cruz, Yang Wu, Henning Ulrich, Alister G. Craig, Célia R.S. Garcia

**Affiliations:** a Department of Physiology, Instituto de Biociências, Universidade de São Paulo, Rua do Matão, travessa 14, n321, CEP 05508-900 São Paulo, SP, Brazil; b Department of Parasitology, Liverpool School of Tropical Medicine, Liverpool, United Kingdom; c Department of Biochemistry, Instituto de Química, Universidade de São Paulo, São Paulo, SP, Brazil

**Keywords:** Malaria, Cytoadhesion, Tumor necrosis factor, *Plasmodium falciparum*, Calcium signaling, Proliferating cell nuclear antigen-1

## Abstract

**Background:**

Plasmodium has a complex biology including the ability to interact with host signals modulating their function through cellular machinery. Tumor necrosis factor (TNF) elicits diverse cellular responses including effects in malarial pathology and increased infected erythrocyte cytoadherence. As TNF levels are raised during *Plasmodium falciparum* infection we have investigated whether it has an effect on the parasite asexual stage.

**Methods:**

Flow cytometry, spectrofluorimetric determinations, confocal microscopy and PCR real time quantifications were employed for characterizing TNF induced effects and membrane integrity verified by wheat germ agglutinin staining.

**Results:**

TNF is able to decrease intracellular parasitemia, involving calcium as a second messenger of the pathway. Parasites incubated for 48 h with TNF showed reduced erythrocyte invasion. Thus, TNF induced rises in intracellular calcium concentration, which were blocked by prior addition of the purinergic receptor agonists KN62 and A438079, or interfering with intra- or extracellular calcium release by thapsigargin or EGTA (ethylene glycol tetraacetic acid). Importantly, expression of PfPCNA1 which encodes the *Plasmodium falciparum* Proliferating-Cell Nuclear Antigen 1, decreased after *P. falciparum* treatment of TNF (tumor necrosis factor) or 6-Bnz cAMP (N^6^-benzoyladenosine-3′,5′-cyclic monophosphate sodium salt).

**Conclusions:**

This is potentially interesting data showing the relevance of calcium in downregulating a gene involved in cellular proliferation, triggered by TNF.

**General significance:**

The data show that *Plasmodium* may subvert the immunological system and use TNF for the control of its proliferation within the vertebrate host.

## Highlights

• TNF is able to decrease parasitemia in *P. falciparum*‐infected RBCs.
• TNF induced rises in intracellular calcium concentration, which were blocked by the purinergic receptor agonists KN62 and A438079.
• Interfering with intra‐ or extracellular calcium release by thapsigargin or EGTA also block TNF‐induce calcium release in *P. falciparum*.
• Expression of the *P. falciparum* Proliferating‐Cell Nuclear Antigen 1 (PfPCNA1) decreased after *P. falciparum* treatment with TNF or 6‐Bnz cAMP.
• The data show that Plasmodium may subvert the immunological system and use TNF for the control of its proliferation within the vertebrate host.

## 1 Introduction

*Plasmodium* has a complex life cycle including the ability to interact with host signals, modulating their function through cellular machinery and membrane receptors. Signal transduction inside *Plasmodium* has been shown as a major mechanism to control parasite development [1], [2], [3]. *Plasmodium* infection remodels the cytoskeleton network in infected erythrocyte (IE) [4] and communication has been shown to occur by exosome-like vesicles sensitive to actin filaments and microtubule inhibitors, which are released into culture supernatant and provide means to respond to environmental changes [5].

Tumor necrosis factor (TNF) is a well-known pro-inflammatory cytokine involved in host immunological responses that elicit diverse cellular responses ranging from proliferation to activation of apoptosis (reviewed in [6], [7], [8] and inhibitors of TNF production reduced IE cytoadherence [9], [10].In mammalian cells, the biological activities of TNF are mediated by two distinct cell-surface receptors: tumour-necrosis factor receptor-1 or 2 (TNFR1 and TNFR2). Ligand binding to TNFR complexes induces intracellular signal transduction. TNF induces an upstream activation of IƘƘB by phosphorylation, ubiquitination and degradation of IƘB alpha. After phosphorylation, NF-ƘB can be released and translocate to the nucleus, where it binds to DNA sequences modulating gene expression [11]. The search for molecular mechanisms involved in the TNF signaling cascade showed its synthesis can be modulated at both transcriptional and translational levels by the p38 MAP kinase pathway [12], [13], [14]. Increasing intracellular levels of cyclic adenosine monophosphate (cAMP) can block TNF synthesis and cause activation of protein kinase A preventing transcription of the gene encoding TNF and decrease in the cytokine synthesis [15].

Receptors and pathways involved in TNF signaling have not been reported in *Plasmodium*. In the host, TNF genotypes are associated with malaria clinical outcomes by changes in the cytokine plasma levels [16] and apoptosis in CD4 T cells might be mediated by TNFR1 death receptor via TNF pathway during *Plasmodium vivax* infection [17].

P2X7 receptor (P2X7R) is involved in signal transmission during the inflammatory response by inducing intracellular calcium rise, activates transcription factors and leads to release of pro-inflammatory cytokines [18], [19] while the presence of putative purinergic receptor was pharmacologically indicated in *Plasmodium* [20], [21].

TNF was also shown to have effects on anti-microbial activities [22] and high levels of this cytokine are associated with malarial pathology [23]. Importantly, *Plasmodium* hepatic development is inhibited by TNF [24], [25] and the effect mediated by Interleukin (IL)-6 in response to TNF stimulation [24], [25].

During inflammation or infections the production of TNF is increased and the present work evaluated if the intra-erythrocytic stage of *Plasmodium falciparum* might also be modulated by this cytokine. Our data show that TNF is able to reduce *P. falciparum* parasitemia through a calcium-cAMP downstream signaling with, possibly, PCNA1 as a target. The cross-talk between calcium and cAMP is a well-known mechanism in mammalian cells and has been reported to occur in *P. falciparum* infected cells [26] as well as *P. chabaudi* [27].

## 2 Material and methods

### 2.1 *P. falciparum* culture

*P. falciparum* parasites (3D7, A4 and ItG lines) were cultured in RPMI 1640 (Invitrogen) supplemented with 37.5 mM HEPES, 7 mM D-glucose, 6 mM NaOH, 25/ml gentamicin sulphate, 2 mM L-glutamine and 10% human serum and maintained in human erythrocytes under a gas mixture of 5% O_2_, 5% CO_2_, and 90% N_2_. Previous to culture the erythrocyte suspensions were washed 3 times in RPMI 1640 for 30 min followed by centrifugation at 8000 rpm. The top supernatant layer was then aspirated to remove lymphocytes and platelets. Cultures were maintained, synchronized by using 5% sorbitol and experiments performed 24 h after synchronization. Procedures were approved by the São Paulo University Ethics Committee.

### 2.2 Spectrofluorimetric determinations of intracellular calcium fluxes

Isolated parasites were obtained by adding saponin (SIGMA) to a final concentration of 0.05% and the mixture was kept on ice for 5 min to lyse the erythrocytes. Following centrifugation at 10,000 rpm at 4 °C for 10 min, erythrocyte ghosts were removed and the free parasite pellets were washed twice using RPMI 1640 for 2 min at 10,000 rpm to remove any insoluble material. Isolated *P. falciparum* (3D7) were incubated for 60 min at room temperature with the fluorescent calcium indicator Fluo-4/AM (5 μM) in MOPS buffer with Ca^2 +^ (2 mM) and 1.8 mM probenecid. The cell suspension was then washed three times [26]. For experiments without calcium, saponin isolated parasites were incubated 1 min with EGTA (2 or 5 mM) in MOPS buffer without calcium. Spectrofluorimeric measurements were performed in a Shimadzu RF-5301 PC at 37 °C with parasites (10^8^ cells ml^− 1^) in a 1 ml cuvette. The fluorescence was measured continuously (acquisition rate: every 0.5 s) for 550 s at 37 °C and TNF (0.25, 0.5, 1 or 10 ng/ml), thapsigargin (10 μM) or control added during time course experiments. Saponin isolated parasites were incubated with KN62 (10 μM) for 30 min or A438079 (5 μM) for 2 min followed by addition of TNF (1 ng/ml). Excitation/emission wavelengths adjusted to 505/530 nm for Fluo4-AM.

### 2.3 Immunofluorescence

Immunofluorescence was performed in saponin isolated *P. falciparum* (3D7) parasites or IE after TNF (1 ng/ml) or PBS treatment for 1 h at 37 °C. Cells were fixed with paraformaldehyde 4% and glutaraldehyde 0.0075% for 30 min at room temperature, washed in PBS 3 times and incubated with DAPI (1:1000 in PBS) and washed 3 more times in PBS. WGA (*Wheat Germ Agglutinin*) (10 μg/ml) was added for 10 min at 37 °C followed by 3 final PBS washes. Slides were sealed with VectaShield (Vector). Image acquisitions were performed on a confocal microscope and LSM Meta software using Plan-NeoFluor 63 × objective. The samples were excited at 585–615 nm and emission at 650 nm.

### 2.4 Invasion assay

Invasion was assessed using flow cytometry analysis in *P. falciparum* line 3D7 incubated for 48 h with TNF (1; 2 or 8 ng/ml) or PBS, and medium replaced every 24 h. IE was then stained with dihydroethidine (5 μg/ml) for 20 min at 37 °C and analysed by dot plots (side scatter versus fluorescence) of 10^5^ cells. Dihydroethidine (DE) was excited with a 488 nm Argon laser and fluorescence emission was collected at 518–605 nm. Parameters subject to adjustment of the FACSCalibur flow cytometer were forward scatter (FSC) (log scale, E-1), SSC (log scale, 269), FL-2 (log scale, 505). For all flow cytometry experiments initial gating was carried out with unstained erythrocytes to account for erythrocyte autofluorescence [28].

### 2.5 PCR real time (RT-PCR)

Total RNA from IE previously incubated for 1 h at 37 °C with 6-Bnz cAMP (20 μM), TNF (1 ng/ml) or PBS for 1 h at 37 °C were extracted by Trizol followed by DNAse I (20 U) treatment for 30 min at room temperature. RNA (0.5–1 μg) were transcribed using random primers and Superscript II RNAase H reverse transcriptase (Invitrogen). PCR was performed using Platinum Taq polymerase (Invitrogen) for 32 cycles and the visualized on agarose gels. Real-time PCR was performed in triplicate using Power SYBR-green mix (Applied Biosystems). The sequences of synthesized primers used in this work are provided in Supplemental material Table 1 (PfPCNA1 and Seryl-tRNA synthetase control) PlasmoDB number PF3D7_1361900 for Proliferating cell nuclear antigen and PF07_0073 to serine — tRNA synthetase). The relative changes in the amount of mRNA of target genes/seryltRNA synthetase were determined by the formula 2^ΔΔct^ or by the threshold cycle in absolute numbers mRNA.

### 2.6 Endothelial cells

Human dermal microvascular endothelial cells (HDMEC) were obtained from commercial providers (Promocell) and maintained in a manufacturer´s recommended medium. Cells at passages 4–7 were used for all experiments. Prior to experimentation cells were stimulated with TNF at a final concentration of 20 ng/ml overnight. This procedure stimulates the induction of ICAM-1 expression on the surface of the endothelial cells. Confluent HDMEC cells were detached from T25 flask using Accutase followed by addition of HDMEC media. Next, cells were centrifuged at 1200 rpm for 3 min and 1.5 × 10^6^ cells per 100 μL used for flow assays.

### 2.7 Flow based endothelial cell assays

Adhesion assays were performed as previously described [29]. Briefly, HDMEC cell suspension was applied to endothelial biochips (Vena8-Cellix) coated with fibronectin (100 μg/ml), and allowed to settle for 3 h at 37 °C to form a confluent monolayer. HDMEC medium was added to biochips every 30 min. A4 mid-trophozoite parasites were prepared at 3% parasitemia and 2% haematocrit and pre-treated with FK506 (1.5 or 6 μM), calyculin (2 or 10 μM), NFKKB inhibitor (1 or 2 μM) or DMSO (0.04%) for 30 min at 37 °C in binding buffer. After flowing binding medium through the microslide for 2 min, prepared parasites were flowed through at a wall shear stress of 0.05 Pa for 5 min, followed by a wash with binding buffer (2 min). Stationary adherent parasites were counted in at least six fields along the slide among 3–5 independent experiments. Results shown are compared to DMSO treated culture.

### 2.8 Static protein binding assays

ICAM-1 or CD36 (50 μg/ml) in PBS was spotted on 60-mm Petri plates for 2 h at 37 °C in a humidified chamber, and used for binding assays with parasite cultures. PBS and BSA were used as negative controls for binding. These plates were then blocked overnight with 1% BSA in PBS at 4 °C. ItG parasites at 3% parasitemia and 1% haematocrit were treated with FK506 (0.375, 0.75 or 1.5 μM) or DMSO (0.04%) for 30 min at 37 °C and added to the spotted plates in binding buffer. The plates were carefully rotated every 10 min for 1 h at 37 °C, and unbound erythrocytes washed away with binding buffer. Bound erythrocytes were fixed in 1% glutaraldehyde for 1h at room temperature and stained with 2% Giemsa for 30 min. Bound IE was counted using a Nikon microscope at 1000 × magnification from six randomly selected distinct fields in triplicate spots from three independent experiments. DMSO treated cultures were used as controls to signify 100% binding.

### 2.9 Quantitative measurement of PfMP-1 levels using BC6 monoclonal Ab

*P. falciparum* (A4 line) synchronized cultures (at least 3% trophozoites) were incubated with FK506 (0.375; 0.75; 1.5 or 6 μM), calyculin (2 or 10 μM), NFKKB inhibitor (1 or 2 μM) or DMSO (0.04%) for 30 min at 37 °C and washed in RPMI 1640 without serum. The pellet was incubated with Plasmagel for enrichment of trophozoites and washed again. Next, parasites were incubated with the BC6 antibody (mouse IgG mAb directed against PfEMP-1), used at a dilution of 1:50 from 310 μg/ml stock in 1% BSA/PBS for 90 min at 37 °C, washed twice in 1% BSA/PBS, centrifuged 2000 rpm for 5 min and the secondary antibody conjugated goat anti-mouse IgG-APC (1:100) (Thermo Scientific) added followed by ethidium bromide 10 mg/ml (1:1000) in 1% BSA/PBS for 90 min at 37 °C. The cells were washed twice in 1% BSA/PBS, centrifuged 2000 rpm for 5 min and fixed in 0.5% paraformaldehyde/PBS or BD CellFix (BD Biosciences) overnight at 4 °C.

For flow cytometry analysis, stained infected cells were diluted in PBS and 50,000 events acquired on a Beckman Coulter FACS XL flow cytometer, gated using forward scatter and side scatter signals to acquire erythrocyte populations excluding debris and FL-1 and FL-2 fluorescence intensity measured. Analysis was performed using FlowJo software. Regions were drawn on density plots of uninfected controls to gate uninfected cells such that < 0.1% of the population fell into the region classed as ethidium bromide positive. This region was then used to create a histogram. Region R1 was created for A4 positive cells based on negative antibody controls, < 0.1% of the ethidium bromide positive population fell into region R1 when no primary antibody was included. The mean fluorescence intensity (MFI) of region R1 was used to quantitate the level of PfEMP-1 on A4 positive infected red blood cells.

### 2.10 Statistical analysis

Results are expressed as mean ± SEM of at least three independent experiments. Student’s t-test was used for comparisons between two groups, whereas for repeated measures ANOVA was used for comparisons among larger groups. P value less than 0.05 were considered indicative of a statistically significant difference. GraphPad Prism software (version 5, San Diego, CA, USA) was used for all statistical tests.

## 3 Results

TNF expression is known to be highly enhanced in *P. falciparum* infection but its role during the asexual stage was not described. The present work showed that TNF is of physiological importance in *Plasmodium*, as it was able to modulate *Plasmodium* intra-erythrocytic invasion, similarly as previously described in the liver stages [25].

**Fig. 1:**
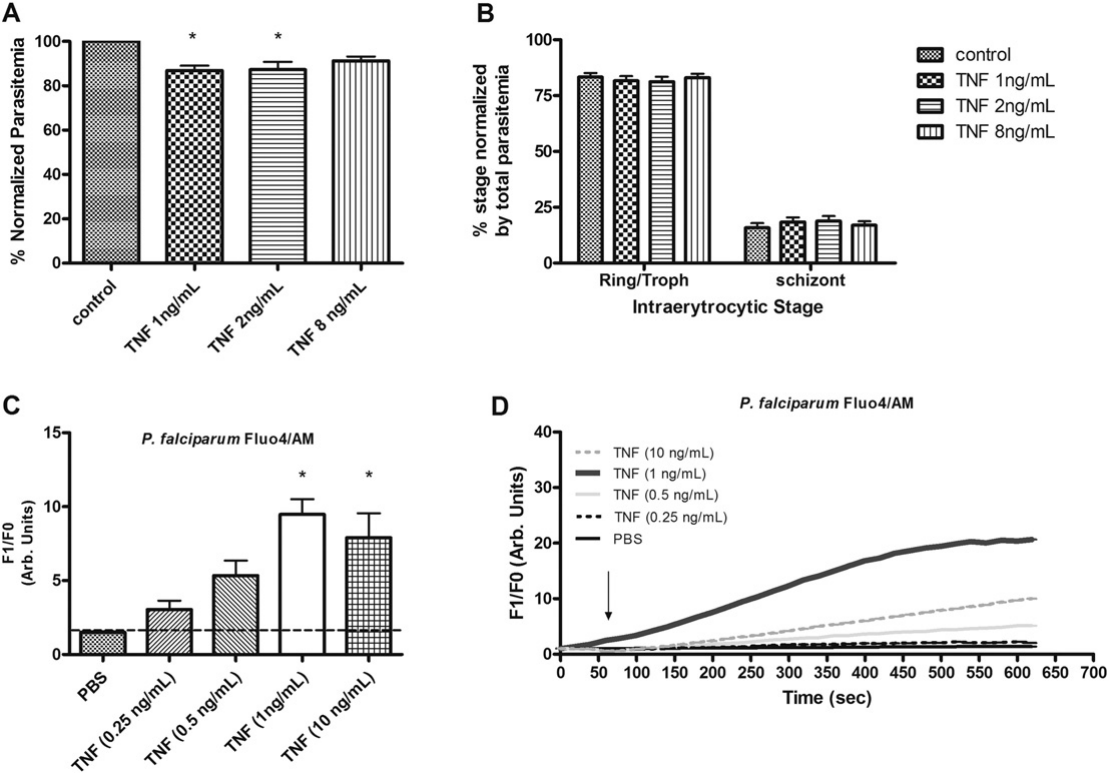
Effects of TNF on *P. falciparum* (3D7) erythrocyte invasion and Ca^2 +^ concentration in isolated parasites. (A) Flow cytometry analyses of parasitemia in synchronized *P. falciparum* (3D7) after 48 h of control or TNF (1, 2 and 8 ng/ml) treatment (* P < 0.05) (B) and intra-erythrocytic stages distribution. Bars represent the number of parasites expressed as percentage of control or ring-trophozoites and schizonts (average) normalized by total parasitemia ± S.E.M. Cells were stained with dihydroethidine (5 μg/ml) and data (10^5^ cells) were compared by one way ANOVA and by Newman–Keuls test. (C) Spectrofluorimetric analyses of Ca^2 +^ concentration in isolated parasites labelled with Fluo4/AM (5 μM) after addition of TNF 0.25; 0.5; 1 or 10 ng/ml (3.05 a.u. ± 0.59, *n* = 11, P = 0.1536; 5.35 a.u. ± 1.01, *n* = 14, P = 0.064; 9.476 a.u. ± 1.03; *n* = 15, P = 0.001 and 7.91 a.u. ± 1.65, *n* = 10, P = 0.034, respectively). P values were calculated by comparison with the PBS data (1.505 a.u. ± 0.07, n = 4). Bar graph means and SEM of at least three independent experiments. Arb. arbitrary. (D) Representative tracing of Fluo4/AM changes over time by addition of TNF (0.25; 0.5; 1 and 10 ng/ml and PBS) in *P. falciparum* isolated parasites.

Parasites where incubated with TNF (1, 2 or 8 ng/ml at a final parasitemia of around 4% (3.98 a.u. ± 0.21, n = 9). We analyzed possible changes in parasitemia and intra-erythrocytic development by flow cytometry (Fig. 1). Data indicate invasion reduction in the presence of 1 and 2 ng/ml TNF treatment with parasitemia being 13.11% (± 5.98 n = 8) and 12.72% (± 9.4 n = 7) lower than untreated control samples. Intra-erythrocytic parasite development was otherwise similar for all other treatments (Fig. 1A and B). Representative histogram showing flow cytometry analysis of total parasitemia and intra-erythrocytic stage distribution can be found at (Supplemental material Fig. S4).

Next we verified the capacity of TNF to modulate calcium signaling in *P. falciparum* parasites. For this purpose, saponin-isolated parasites were incubated with Fluo-4/AM and spectrofluorimetric analyses showed that TNF can increase free intracellular calcium concentration ([Ca^2 +^]_i_) (Fig. 1C and D) with the highest transient increase observed at 1 ng/ml. These quantities reflect typical values used in *in vitro* systems but are towards the higher end of the physiological levels encountered in the human host where TNF concentrations up to 1ng/ml can be found in the plasma of patients with severe disease [30], [31].

**Fig. 2:**
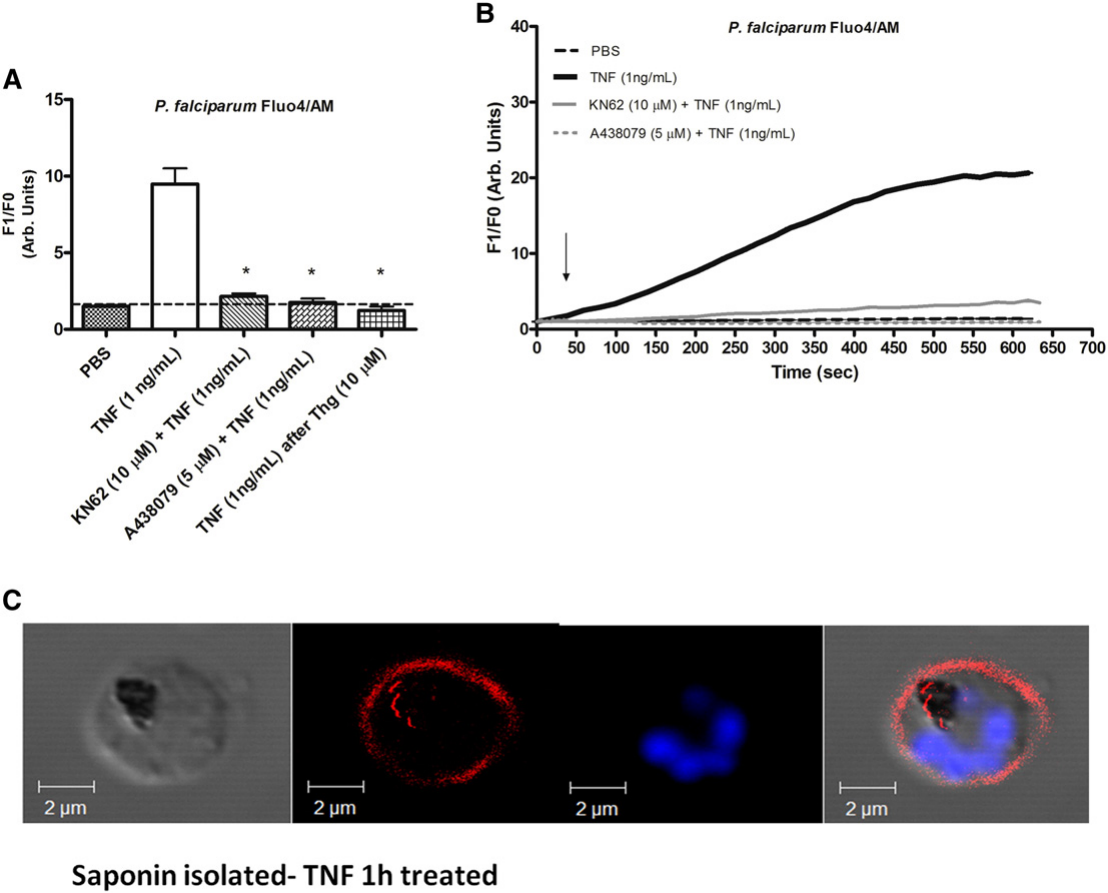
TNF intracellular calcium signalling can be blocked by P2X_7_ antagonist in *P. falciparum* isolated parasites labelled with Fluo4/AM (5 μM). (A) Analyses of Ca^2 +^ concentration after incubation with the purinergic inhibitor KN62 (10 μM) for 30 min, A438079 (5 μM) for 2 min or thapsigargin (10 μM) all followed by addition of TNF (1 ng/ml) (2.15 a.u. ± 0.19, *n* = 13, P < 0.0001; 1.76 a.u. ± 0.24, *n* = 11, P < 0.0001; or 1.236 a.u. ± 0.25; *n* = 11, P < 0.0001, respectively). *P* values were calculated by comparison with the TNF (1 ng/ml) data (9.746 a.u. ± 1.03, *n* = 15). Bar graph means and SEM of at least three independent experiments. Arb. arbitrary. (B) Representative tracing of Fluo4/AM changes over time by addition of PBS, TNF (1 ng/ml) and TNF (1 ng/ml) after incubation with KN62 (10 μM) or A438079 (5 μM) (C) Imaging of wheat germ agglutinin (WGA) staining in *P. falciparum* (*3D7*) *saponin isolated* parasite after TNF (1 ng/ml) treatment for 1 h at 37 °C. Smears are stained with WGA (10 μg/ml for 10 min at 37 °C) and DAPI (1:1000 in PBS 15 min at RT) and observed by confocal microscopy.

The use of kinase inhibitors has been explored for the control of inflammation [15], [32] and the inhibitor KN62 was previously shown to modulate *Plasmodium* pathways [20], [21]. Interestingly KN62 is a Ca^2 +^/calmodulin-dependent protein kinase II inhibitor and P2X_7_ antagonist that also blocked [Ca^2 +^]_i_ increase after TNF addition (Fig. 2A & B), indicating the involvement of the pathways in TNF signaling. Next A438079, a competitive P2X_7_ receptor antagonist also inhibited the TNF/ IE response (Fig. 2A & B). Membrane integrity after TNF treatment was studied following staining isolated parasites with WGA by confocal microscopy imaging, revealing no effects of this factor on membrane integrity, as WGA localization in the membrane was the same for TNF-treated and control samples (Fig. 2C and Supplemental data S1 and S2).

*Plasmodium* intracellular calcium homeostasis is known to be regulated by endoplasmic reticulum (ER) among other organelles [33], [34]. Here we showed using thapsigargin (THG) that the organelle is essential for the calcium response elicited by TNF as there is no response after ER calcium depletion by THG (Fig. 2A & B).

**Fig. 3:**
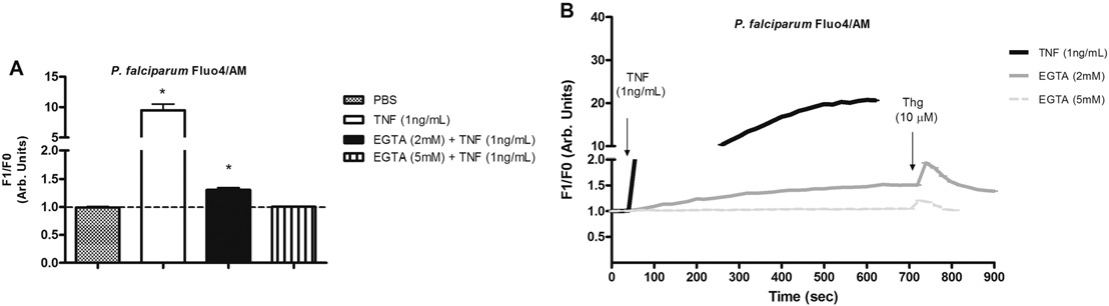
Extracellular calcium is required for TNF signalling in isolated *P. falciparum* labelled with Fluo4/AM (5 μM). (A) Analyses of Ca^2 +^ concentration after addition of TNF (1 ng/ml) (9.746 a.u. ± 1.03, *n* = 15) or incubation with the extracellular calcium chelator EGTA (5 mM) for 1 min followed by addition of TNF (1 ng/ml) (1.002 a.u. ± 0.005, *n* = 11, P = 0.371), EGTA (2 mM) (1.304 a.u. ± 0.038, *n* = 9, P = 0.001) or PBS treatment (0.989 a.u. ± 0.016, n = 3). Bar graph means and SEM of at least three independent experiments. Arb. arbitrary. (B) Representative tracing of Fluo4/AM changes over time by addition of TNF (1 ng/ml) or TNF (1 ng/ml) followed by thapsigargin (10 μM) after incubation with EGTA (2 and 5 mM, respectively).

Extracellular calcium can also be involved in intracellular ion homeostasis so parasites were incubated with EGTA (2 or 5 mM). This was capable of decreasing the calcium ion rise at a concentration of 2 mM, and completely abolished at 5 mM (Fig. 3). The parasite viability during the experiment, in terms of its ability to respond, following THG addition was confirmed at the end of the experiment (Fig. 3).

**Fig. 4:**
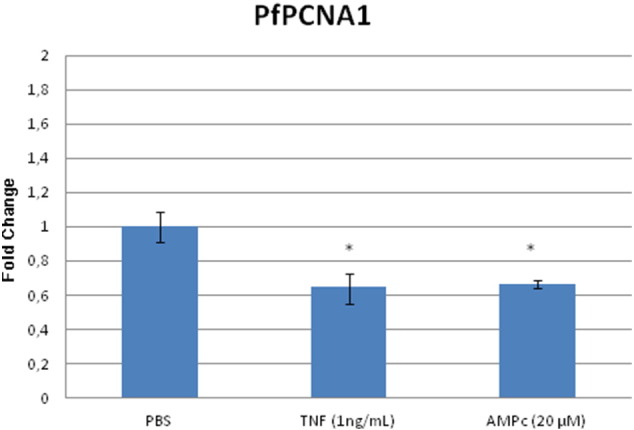
Expression of mRNA in synchronized trophozoites treated with TNF and 6-Bnz cAMP in PfPCNA1. Real Time PCR for *P. falciparum* (3D7) control (PBS), TNF (1 ng/ml) or 6-Bnz-cAMP (20 mM), after incubation for 1 h at 37 °C. Bars represent mean ± S.E.M. in 3 independent experiments (P < 0.05*).

As TNF reduces parasitemia we have searched for a target that might be involved in the control of cell cycle proliferation. A possible target involved in the TNF response could be *P. falciparum* Proliferating-Cell Nuclear Antigen 1 (PfPCNA1), which may be differentially phosphorylated by TNF treatment [35]. We have performed quantitative PCR to verify the expression of PfPCNA1. Since TNF increases cytosolic calcium concentration in *P. falciparum* and calcium is well known to cross talk with cAMP within several cells including malaria parasites [26], [27] we pre-treated *P. falciparum* not only with TNF but also with 6-Bnz cAMP. According to Fig. 4, *P. falciparum* synchronized trophozoites treated with TNF (1 ng/ml) or 6-Bnz cAMP (20 μM) have a decrease in PfPCNA1 mRNA expression in comparison to control treatments (PBS). The data point to PfPCNA1 as a downstream target of the TNF–calcium–cAMP signaling pathway, perhaps for reducing cell cycle proliferation, but more work is needed to confirm this. Other candidates studied (e.g. PfRACK) showed no change upon TNF treatment (Supplemental data — Fig. S3).

It is well established that inhibitors of TNF production reduced IE cytoadherence [9], [10]. As FK506 is a potent suppressor of inflammation and the endothelial cell receptor ICAM-1 expression is increased by pro-inflammatory cytokines [36], we have analyzed if FK506 inhibitor would affect the *P. falciparum* binding under flow and static conditions to HDMEC.

**Fig. 5:**
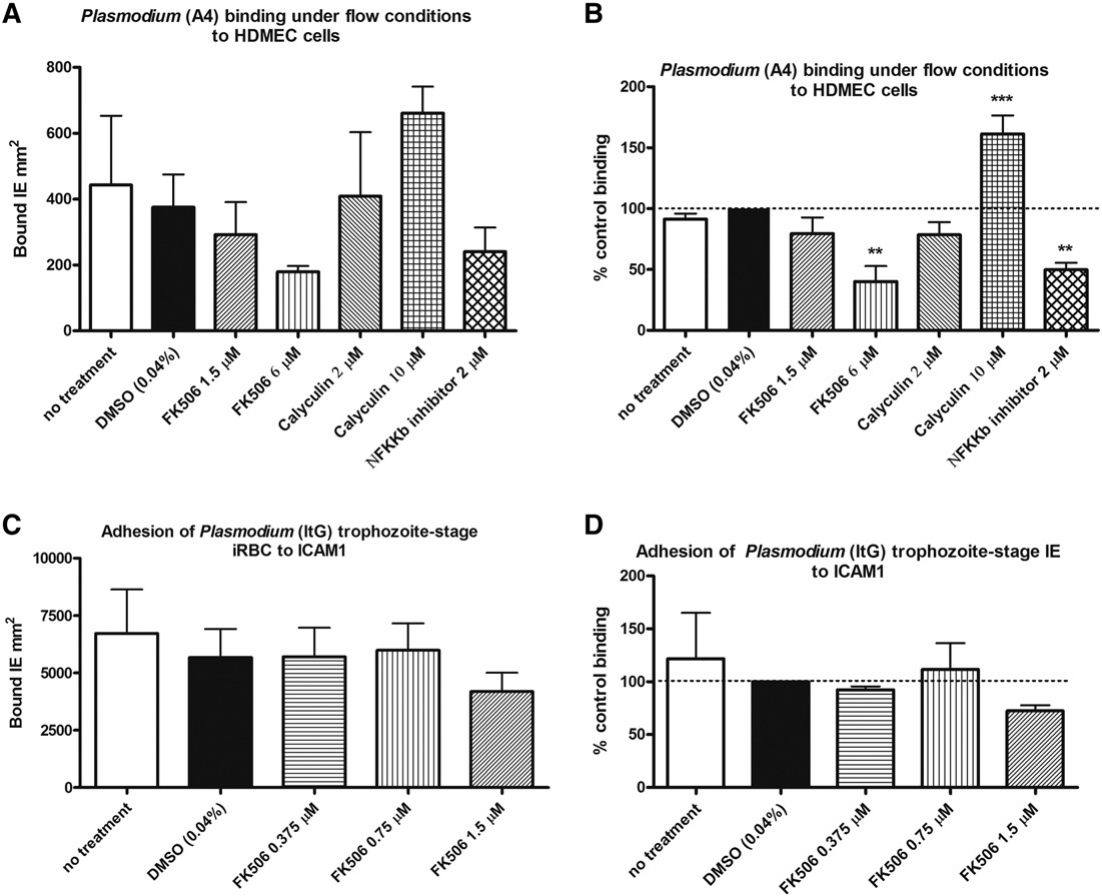
Effects of FK506 in *Plasmodium*-endothelial cell binding under flow and static conditions. (A) Adhesion of trophozoite-stage A4 IE after FK506 (1.5 or 6 μM), calyculin (2 or 10 μM), NFƘƘb inhibitor (2 μM), DMSO (0.04%) or no treatment of parasites under flow assay showing bound IE per mm^2^ or (B) percentage of control (DMSO treated) binding to human dermal microvascular endothelial cells (HDMEC). (C) Adhesion of trophozoite-stage ItG IE to protein ICAM-1 after FK506 (0.375, 0.75 or 1.5 μM), DMSO (0.04%) or no treatment under static conditions to ICAM1 showing bound IE per mm^2^ or (D) percentage of control (DMSO treated) binding. Bars represent mean ± S.E.M. Note differences in A4 erythrocyte binding among 3–5 independent experiments (P < 0.05 *, P < 0.01 **, P < 0.001 ***) under flow condition and no difference observed in ItG erythrocyte binding among three independent treatments (P > 0.05) under static condition.

Fig. 5(A and B) shows the results of experiments performed under flow conditions where parasites were first treated with FK506 (1.5 and 6 μM), calyculin (2 and 10 μM) or Nfkkb inhibitor (2 μM). Higher concentration of calyculin (10 μM) induced an increase in parasite binding indicating the involvement of phosphatases in cytoadherence. While at higher concentration FK506 (6 μM) and Nfkkb inhibitor (2 μM) showed a decrease in binding to HDMEC and highlight the potential importance of the host immune response on parasite signaling and sequential binding to endothelial cells. Under static protein assays (Fig. 5C and D) reduction in binding was not statistically different between FK506 at lower concentrations (0.375, 0.75 or 1.5 μM) compared to the controls, DMSO (0.04 %) or untreated parasites.

**Fig. 6:**
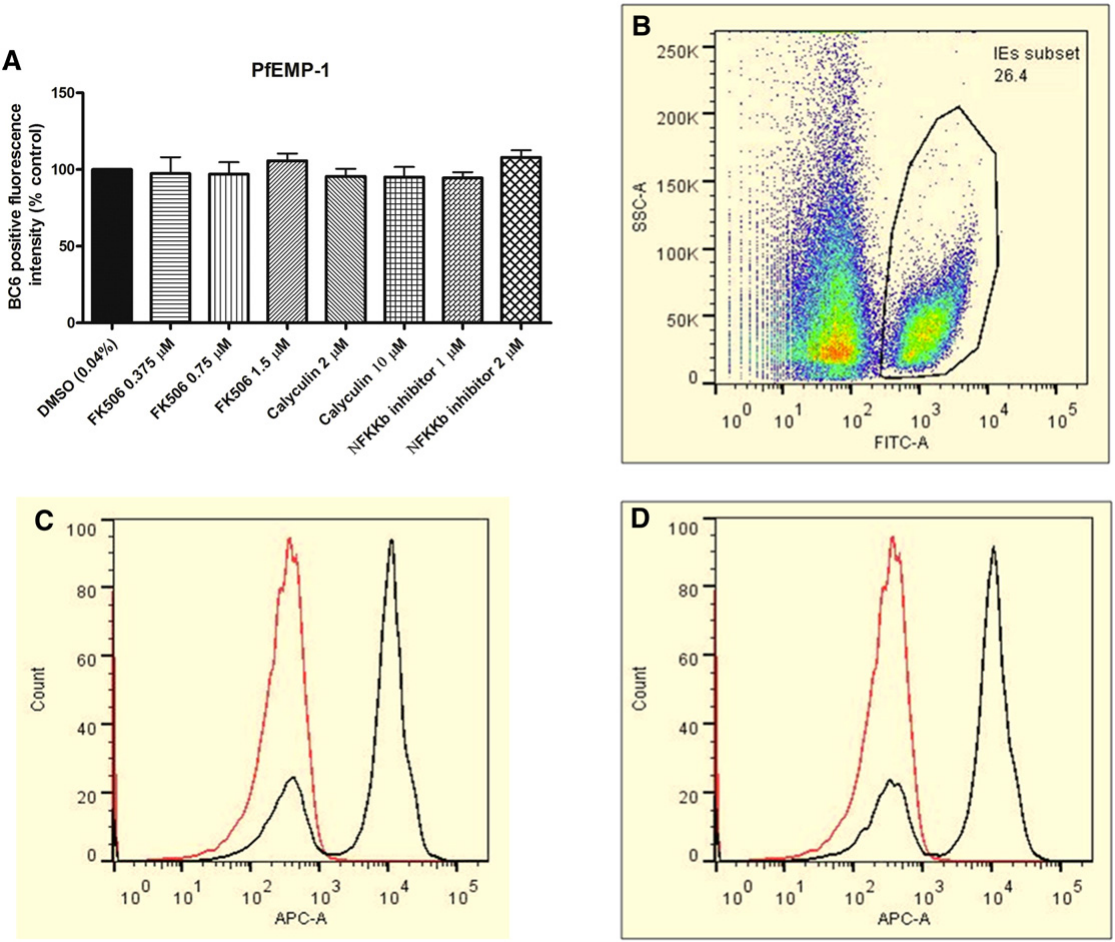
Analysis of PfEMP-1 expression on the surface of live trophozoite-stage A4 IE using BC6 antibody (A) Quantitation of PfEMP-1 levels by flow cytometry analysis after BC6 primary antibody (Alexa 488 coupled secondary antibody) and counterstaining infected erythrocyte with ethidium bromide. (B) Density plots of uninfected erythrocyte population in the left and box IE (infected erythrocyte) representing BC6 positive erythrocytes (gated as ethidium bromide positive) (C) Representative graph showing quantitation of BC6 positive fluorescence intensity (X-axis APC-A) of the infected population without BC6 for negative control (red line); with BC6 and treated with DMSO 0.04% (black line) or (D) FK506 1.5 μM (black line) treated A4 trophozoites. Bars represent mean fluorescence intensity for PfEMP-1 positive population ± S.E.M. for 5 independent experiments. Note no difference in erythrocyte PfEMP-1 quantitation levels (P > 0.05).

To determine whether the ability of parasites (treated as described in Fig. 5) to adhere was due to PfEMP-1 expression on the infected erythrocyte membrane we performed flow cytometry measurements using mAb BC6, specific for a surface epitope of the PfEMP-1 ITvar14 (A4var) variant [37]. The results clearly demonstrate that PfEMP-1 expression is not changed under the treatments (Fig. 6).

## 4 Discussion

TNF is known to be involved in immunological response and required for defense against infectious diseases [38]. During *Plasmodium* inflammation TNF concentration in the serum increases at pmol range, specifically reported in patients with *P. falciparum*, *P. vivax* malaria infection and healthy controls that average plasma TNF levels are 0.027 ng/ml, 0.044 ng/ml and 0 ng/ml, respectively [39].

More recently plasma TNF level was determined by enzyme-linked immunosorbent assay in malaria naive donors and *P. vivax* infected donors indicating its concentration increases in infected samples up to about 1000 pg/ml [17]. The difference in TNF plasma concentration among patients can be due to host genetic alterations which influence levels of inflammatory mediators [16]. We examined the effect of extracellular TNF concentration in modulating *P. falciparum* intracellular development and its molecular mechanism of action. TNF receptors are well characterized in mammalian cells but not described in *Plasmodium* [15]. The effects of recombinant TNF in murine malaria parasites has been studied since 1987 and results *in vitro* and *in vivo* already showed reduced parasitaemia and prolonged survival in mice infected. The authors indicated that TNF acts thought a host cell pathway [40].

The present work showed that TNF is of physiological importance in *Plasmodium* as it was able to modulate *Plasmodium* intra-erythrocytic invasion and to increase [Ca^2 +^]_i_ with the highest effect observed at TNF 1 ng/ml, indicating sensitivity to this cytokine (Fig. 1).

Ion homeostasis and the presence of a classical endo-sarcoplasmic reticulum Ca^2 +^ ATPase which can be blocked by THG causing calcium release and reversible mitochondrial calcium increase is well reported including *Plasmodium* [33], [41], [42], [43]. Thus the importance of external and intracellular calcium stores in TNF pathways was also highlighted by experiments with addition of THG or EGTA incubation, as both inhibited the [Ca^2 +^]_i_ rise due to TNF (Figs. 2 and 3).

Increase in cytoplasmic calcium was previously shown to synchronize *Plasmodium* cell cycle [44] and the cAMP analogue (adenosine 3,5-cyclic monophosphate N6-benzoyl (6-Bz-cAMP) caused alteration of the parasite cell cycle, possibly dependent on PKA activation [26]. These findings suggest that the TNF response requires a tightly controlled calcium dependent pathway.

The ability of the parasite to use calcium for signaling and to sense the environment was linked to the process of *Plasmodium* synchronicity and host circadian rhythms [45] and verified by experiments in the presence of melatonin, other products of tryptophan catabolism and melatonin degradation indicating the presence of a PLC-IP3 mechanism evoked by calcium mobilization [46], [47], [48], [49] and described a “capacitative calcium entry” mechanism to replenish internal calcium pools in *Plasmodium* [50].

To better understand this TNF pathway in parasites the increase in intracellular calcium was analyzed after incubation with the purinergic P2X7 receptor antagonists KN62 and A438079. Interestingly, both compounds inhibited [Ca^2 +^]_i_ elevations induced by TNF (Fig. 2). Indeed the P2X7 receptor has been identified as a down-stream target of TNF actions, shown in the work of Castrichini et al. [19], exploring the pathophysiology of this purinergic receptor in Behçet's disease [19]. The authors of this work showed that incubation of monocytes resulted in an increase of P2X7 receptor expression and Bz-ATP calcium influx. In view of that, the TNF-triggered calcium fluxes are supposed indirect actions, involving TNF-induced P2X7 receptor activation, which then opens receptor channels for calcium flux. Mammalian cells are described to form P2X1–P2X7 ATP-gated ion channels permeable to Ca^2 +^ among other ions, while the P2X7 receptor intracellular domain couple receptor activation to intracellular signaling pathways [51], [52], [53]. In *Plasmodium* it was previously shown that KN62 inhibited *P. falciparum* invasion and [Ca^2 +^]_i_ rises due to stimulation by extracellular ATP [21], and in *P. berghei* KN62 altered profiles of merozoite surface protein 1 processing and cell cycle progress [20]. Changes in [Ca^2 +^]_i_ also elicited cysteine proteases activity in the parasite [34], [54]. Previous work has already suggested inter-relationships for TNF-P2X7 receptor signaling in other cellular contexts, such as P2X7 receptor expression modulation in immune cells in Behçet's disease [19] or the P2X7 receptor-dependent regulation of TNF release in microglial cells [18] (Further work will elucidate the context of P2X7 receptor–TNF interactions in the biology of *P. falciparum.*

Interestingly expression of PfPCNA1, which encodes the proliferating cell nuclear antigen-1, a key factor for DNA polymerase on the leading strand and plays accessory roles in DNA replication/repair [55], [56], [57], decreased after treatment with TNF (1 ng/ml) or 6-Bnz cAMP (20 μM) indicating PfPCNA1 as a potential target of the pathway.

Experiments to verify the influence of immune/ inflammation response to parasite cytoadherence indicated a change in binding to HDMEC after treating parasites with the immunosuppressant FK506 or Nfkkb inhibitor. Results highlighted the importance of host immune response on parasite signalling, providing an important link that needs to be better characterized in further studies.

**Fig. 7:**
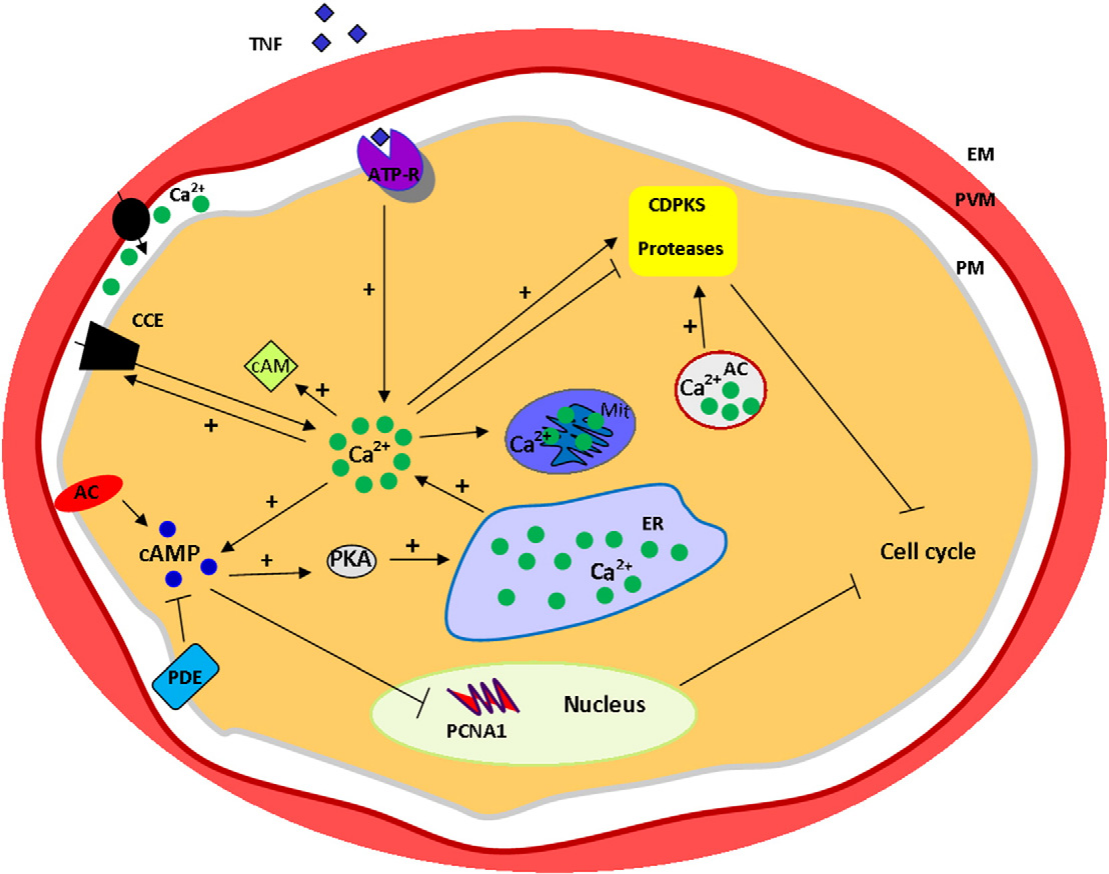
Schematic model for the molecular mechanism of TNF action by calcium signaling on *Plasmodium* inhibition of intraerythrocytic cell cycle. Extracellular TNF (tumor necrosis factor) binds and modulates a putative ATP receptor (ATP-R) at the parasite membrane (PM), leading to intracellular calcium rise. The parasite has mechanisms to deal with calcium homeostasis such as: endoplasmic reticulum (ER), mitochondria (Mit) and acidic pools (AC) or via influx of extracellular calcium by the capacitative calcium entry (CCE). Cyclic adenosine monophosphate (cAMP) is able to promote further calcium increase from the ER while its concentration in the cytosol is regulated by Ca^2 +^, adenylyl cyclase (AC) and phosphodiesterase (PDE). Ca^2 +^ concentration in the parasite cytosol modulates several downstream effectors such as calmodulin (cAM), calcium-dependent protein kinases (CDPKs), proteases activation and possibly targeting gene expression (such as PCNA1) which can act by inhibiting the intracellular parasite cell cycle.

A possible hypothesis might be that TNF is inducing intracellular calcium increase, which includes molecules targeting upstream signalling events involved in cellular responses by the parasite. The proteins transducing these signals to the parasite nucleus are not known, although published data from our group [35] suggests that phosphorylation of a sub-population of PfPCNA1 may be involved. Our pathway hypothesis is shown in Fig. 7.

The present work indicates that intracellular calcium concentration can be modulated by external TNF treatment of the IE and the pathway’s biological importance indicated by the modulation of *P. falciparum* erythrocyte invasion. Moreover, our data shows that *Plasmodium* subverts the immunological system and uses TNF for the control of its proliferation within the vertebrate host.

The following are the Supplementary data related to this article.

Fig. S1 Imaging of wheat germ agglutinin (WGA) staining along *P. falciparum 3D7* intracellular cell cycle after TNF (1 ng/mL) treatment for 1h at 37 °C. Smears are stained with WGA (10 μg/mL for 10 min 37 °C) and DAPI (1:1000 in PBS 15 min RT) and observed by confocal microscopy. Fig. S1

Fig. S2 Imaging of wheat germ agglutinin (WGA) staining along *P. falciparum 3D7* intracellular cell cycle after PBS treatment for 1 h at 37 °C. Smears are stained with WGA (10 μg/mL for 10 min 37 °C) and DAPI (1:1000 in PBS 15 min RT) and observed by confocal microscopy. Fig. S2

Fig. S3 Expression of mRNA in synchronized trophozoites treated with TNF and 6-Bnz cAMP in PfRACK. Real Time PCR for *P. falciparum* (3D7) control (PBS), TNF (1 ng/mL) or 6-Bnz-cAMP (20 mM), after incubation for 1 h at 37 °C. Bars represent mean ± S.E.M. in 3 independent experiments (P < 0.05*). Fig. S3

Fig. S4 Representative histograms showing flow cytometry analysis of total parasitemia and intra-erythrocytic stage distribution (ring-trophozoites or schizonts) in synchronized *P. falciparum* (3D7) infected erythrocyte after 48 h treatment of (A) uninfected erythrocytes (RBC non), (B) IE without dihydroethidine (DE), (C) control (PBS), (D and E) TNF (1 and 2 ng/mL, respectively) and F) overlayed histogram (blue, brown, red line, black and gray line, respectively). Gates presented here were used to define the mono (ring-trophozoites) — and multinucleated (schizonts) populations of the parasite and total parasitemia. Dihydroethidine was excited with a 488 nm argon laser and fluorescence emission collected at 518–605 nm. Fig. S4

Supplemental Table 1 Primers used in RT-PCR experiments. Supplemental Table 1

## Conflict of interest statement

The authors confirm that there are no conflicts of interest.

## Transparency Document

Transparency document. Image 1
