# Supplementary figures and images for: Tumor necrosis factor reduces Plasmodium falciparum growth and activates calcium signaling in human malaria parasites
Source: Biochim Biophys Acta. 2016 Jul;1860(7):1489–97. doi: 10.1016/j.bbagen.2016.04.003 (PMC4876768; doi:10.1016/j.bbagen.2016.04.003)

## Supplemental data

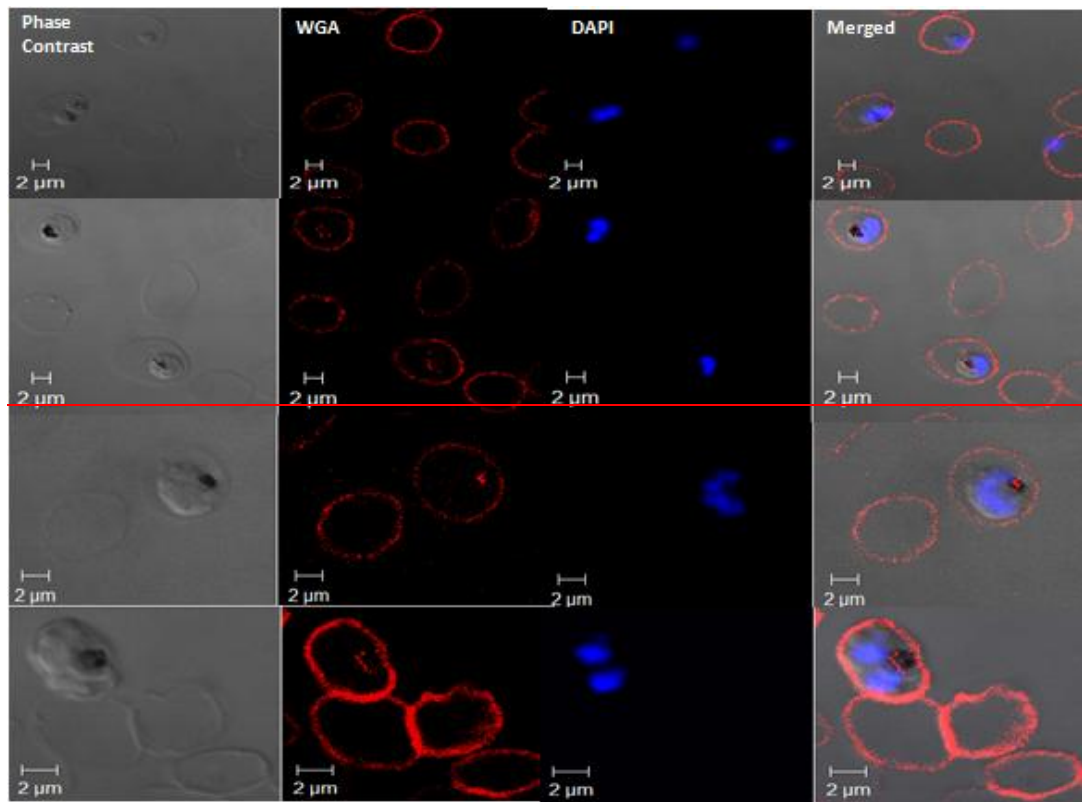

Figure S1

Supplement: Fig. S1 — Imaging of wheat germ agglutinin (WGA) staining along P. falciparum 3D7 intracellular cell cycle after TNF (1 ng/mL) treatment for 1h at 37 °C. Smears are stained with WGA (10 μg/mL for 10 min 37 °C) and DAPI (1:1000 in PBS 15 min RT) and observed by confocal microscopy. [file mmc1.pdf]

## Supplemental data

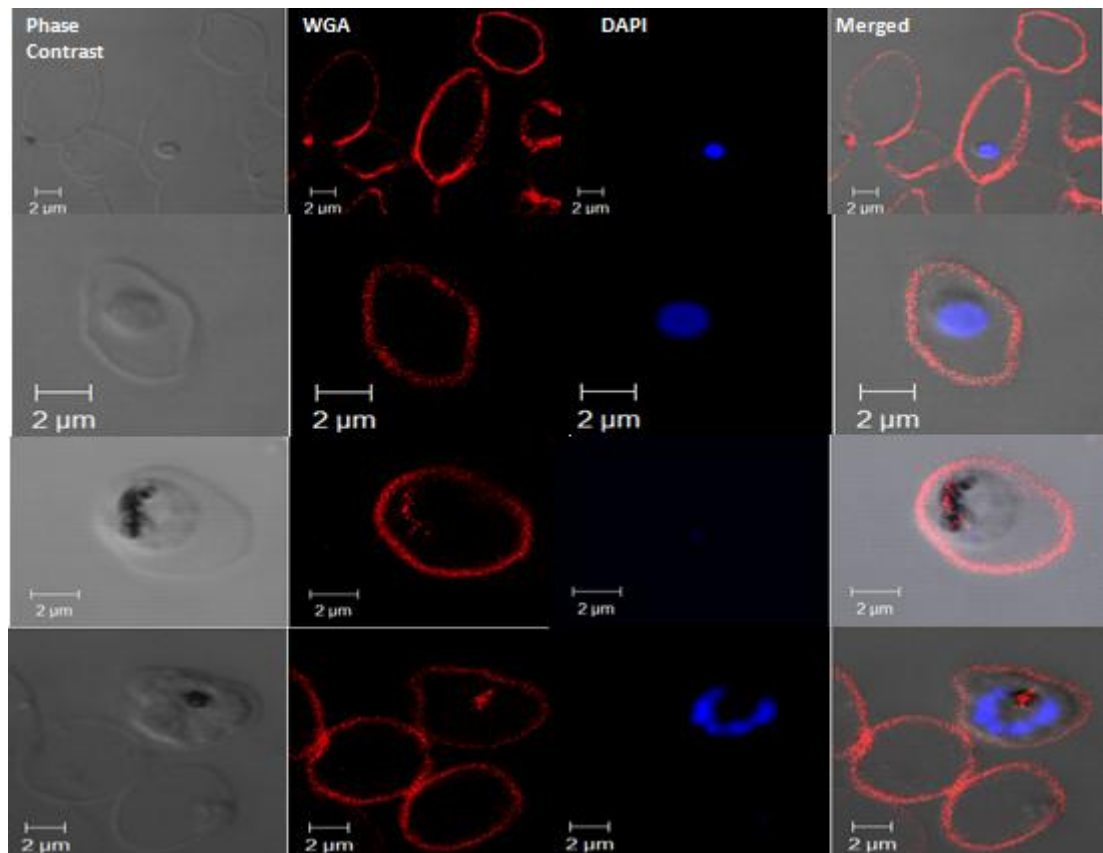

Figure S2

Supplement: Fig. S2 — Imaging of wheat germ agglutinin (WGA) staining along P. falciparum 3D7 intracellular cell cycle after PBS treatment for 1 h at 37 °C. Smears are stained with WGA (10 μg/mL for 10 min 37 °C) and DAPI (1:1000 in PBS 15 min RT) and observed by confocal microscopy. [file mmc2.pdf]

## Supplemental data

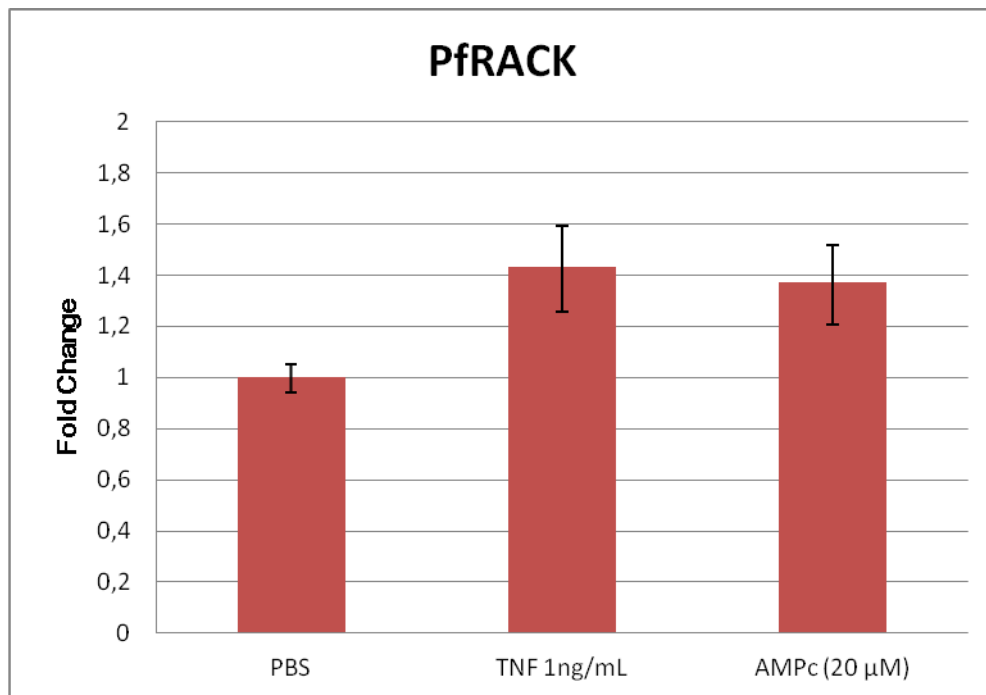

Figure S3

Supplement: Fig. S3 — Expression of mRNA in synchronized trophozoites treated with TNF and 6-Bnz cAMP in PfRACK. Real Time PCR for P. falciparum (3D7) control (PBS), TNF (1 ng/mL) or 6-Bnz-cAMP (20 mM), after incubation for 1 h at 37 °C. Bars represent mean ± S.E.M. in 3 independent experiments (P < 0.05*). [file mmc3.pdf]

## Supplemental data

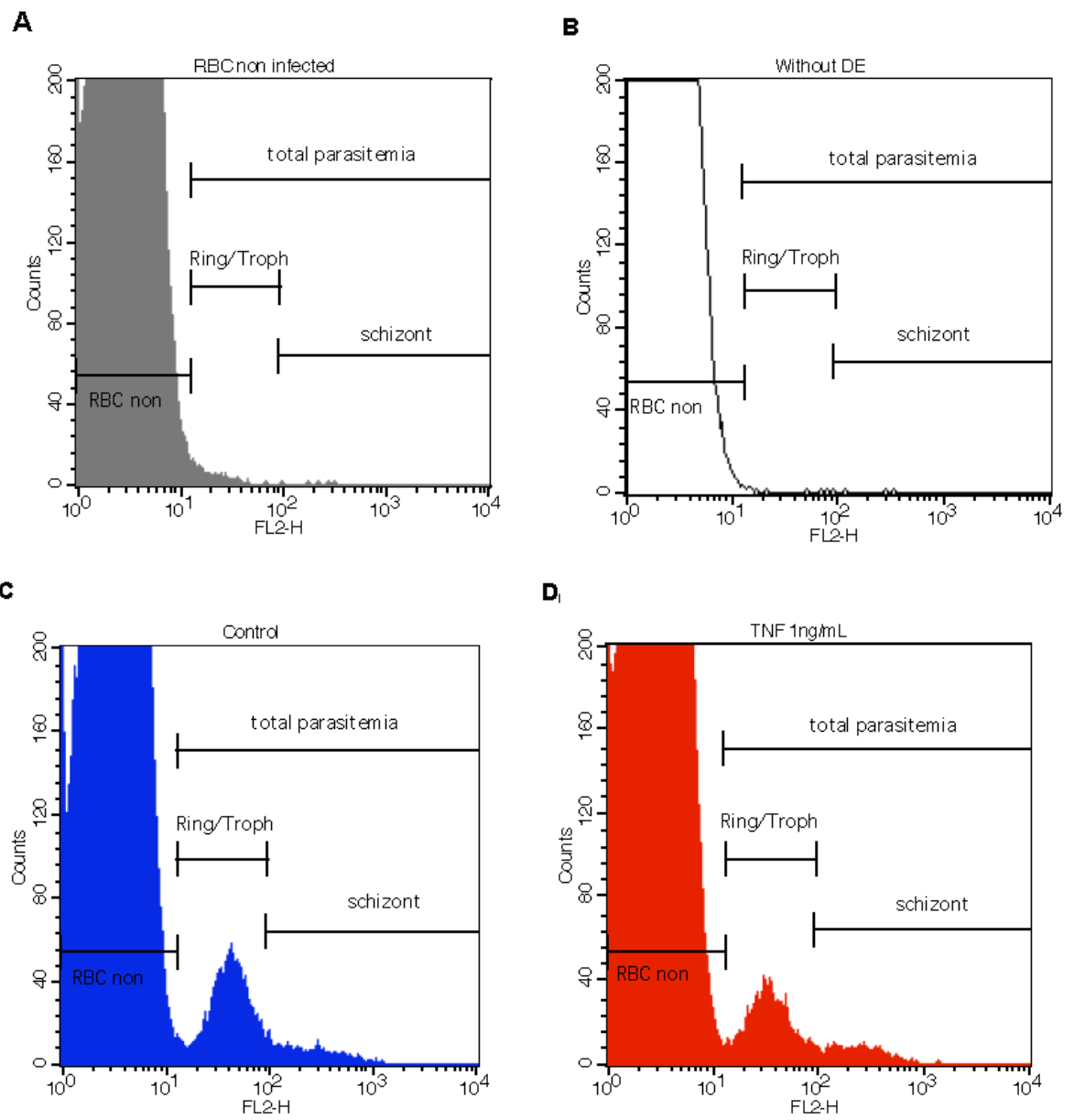

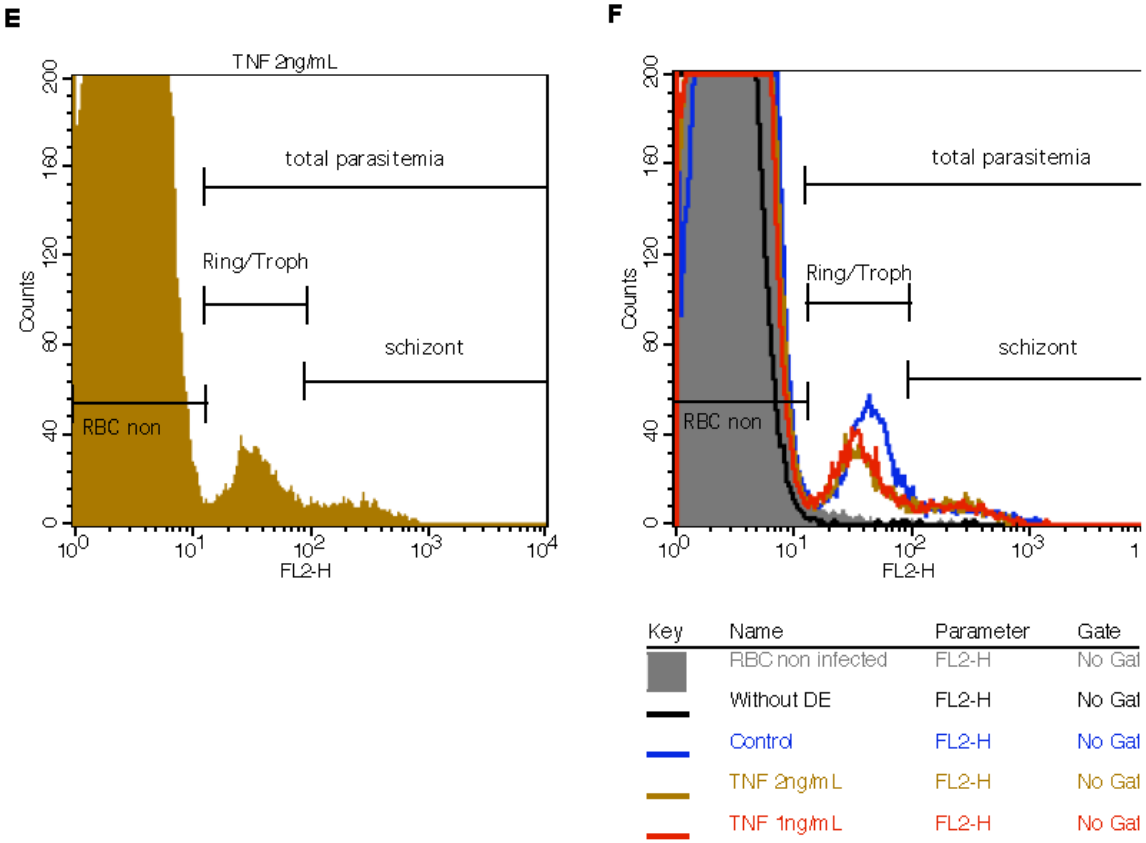

Figure S4

Supplement: Fig. S4 — Representative histograms showing flow cytometry analysis of total parasitemia and intra-erythrocytic stage distribution (ring-trophozoites or schizonts) in synchronized P. falciparum (3D7) infected erythrocyte after 48 h treatment of (A) uninfected erythrocytes (RBC non), (B) IE without dihydroethidine (DE), (C) control (PBS), (D and E) TNF (1 and 2 ng/mL, respectively) and F) overlayed histogram (blue, brown, red line, black and gray line, respectively). Gates presented here were used to define the mono (ring-trophozoites) — and multinucleated (schizonts) populations of the parasite and total parasitemia. Dihydroethidine was excited with a 488 nm argon laser and fluorescence emission collected at 518–605 nm. [file mmc4.pdf]
