# Supplementary material for: Tumor necrosis factor reduces Plasmodium falciparum growth and activates calcium signaling in human malaria parasites
Source: Biochim Biophys Acta. 2016 Jul;1860(7):1489–97. doi: 10.1016/j.bbagen.2016.04.003 (PMC4876768; doi:10.1016/j.bbagen.2016.04.003)
Supplement: Supplemental Table 1 — Primers used in RT-PCR experiments. [file mmc5.doc]

| Primer | | Sequence |  | |
| --- | --- | --- | --- | --- |
| Seryl-tRNA sintetase-FW | TGGAACAATGGTAGCTGCAC | | |  |
| Seryl-tRNA sintetase-RV | TCATGTATGGGCGCAATTT | | | |
| Pf3D7 PCNA- FW | TGTGATCGTGAAAGAGTTTTAGG | | | |
| Pf3D7 PCNA- RV | TGCATCAAATCCTTCTTCACA | | | |
| PfRACK- FW | TCAGGTTCTTGGGACCATTC | | | |
| PfRACK- RV | TGGTGAAGGTGAAAATCGAA | | | |

**Supplemental Table 1** - Primers used in RT-PCR experiments
